# Effect of non-invasive neuromodulation techniques on vascular cognitive impairment: A Bayesian network meta-analysis protocol

**DOI:** 10.1371/journal.pone.0284447

**Published:** 2024-01-04

**Authors:** Long Yan, Linna Wu, Hong Li, Yulin Qian, Meng Wang, Yu Wang, Baomin Dou, Tao Yu

**Affiliations:** 1 First Teaching Hospital of Tianjin University of Traditional Chinese Medicine, Tianjin, P. R. China; 2 National Clinical Research Center for Chinese Medicine Acupuncture and Moxibustion, Tianjin, P. R. China; 3 Tianjin University of Traditional Chinese Medicine, Tianjin, P. R. China; Stanford University, UNITED STATES

## Abstract

**Background:**

VCI is a severe public health problem facing the world today. In addition to pharmacological treatment, non-invasive neuromodulation techniques have also been effective. At this stage, non-invasive neuromodulation techniques combined with pharmacological treatment are the mainstay of clinical treatment, and clinical trials are continuing to be conducted, which is becoming the direction of treatment for VCI. Therefore, we outline this systematic review and network meta-analysis protocol to evaluate and rank clinical data in future studies which can develop optimal protocols for the clinical treatment of VCI with non-invasive neuromodulation techniques in combination with drugs.

**Methods:**

The network meta-analysis will search eight databases, including PubMed, Embase, Cochrane Library, Web of Science, China Knowledge Infrastructure Library (CNKI), China Biology Medicine disc (CBM)), Wanfang Data Knowledge Service Platform and Vipshop Journal Service Platform (VIP), for a period of from the establishment of the library to January 30 2022. The quality of the studies will be evaluated using the Cochrane Review’s Handbook 5.1 and the PEDro scale to assess the evidence and quality of the included randomised controlled trials. Risk of bias assessment and heterogeneity tests will be performed using the Review Manager 5.4 program, and Bayesian network meta-analysis will be performed using the Stata 16.0 and WinBUGS 1.4.3 program.

**Results:**

The results of the network meta-analysis will be published in a peer-reviewed journal.

**Conclusions:**

Our study is expected to provide high quality evidence-based medical evidence for the treatment of VCI by clinicians.

**Trial registration:**

**PROSPERO:** CRD42022308580.

## Introduction

Vascular cognitive impairment (VCI) is an umbrella term for a range of cognitive impairments primarily caused by cerebrovascular disorders [1, 2], generally considered to include mild VCI, vascular dementia (VaD), and vascular-related Alzheimer’s disease (AD) [3, 4]. The three categories of VCI are cognitive impairment following stroke and cognitive impairment caused by invisible cerebrovascular lesions that can only be detected by autopsy or imaging [5]. The main clinical manifestations of VCI have impaired thinking and executive abilities, including memory, behavioural and emotional dysfunctions, and other neurological deficits such as dysarthria(difficulty with speech), parkinsonism and reflex asymmetry [6]. According to statistics, VCI is the second leading cause of dementia worldwide after AD, accounting for approximately 20% of people with dementia [7]. Modern autopsy studies show that the risk of vascular brain injury accounts for 33% of dementia [8]. The primary pathological basis of VCI is a deficiency of cholinergic transmitters in patients [9], and clinical drug therapy is mainly based on acetylcholine inhibitors. The main drugs approved by the US FAD for VCI treatment are Donepezil, Rivastigmine and Galantamine, and the first two of which have been recommended as Level A evidence [10]. Other drugs commonly used in Vad treatment include Memantine, Citicoline, Cilostazol, Naftidrofuryl, Sertraline, and Vinpocetine [9]. However, the clinical evidences of these drugs are currently insufficient. In terms of neurological mechanisms, VCI patients’ recovery depends mainly on neuroplasticity which is the functional rebuilding of the central nervous system, especially the cortex, following injury and stimulation. Neuroplasticity relies on the regeneration of brain tissue vasculature and the enhancement of synaptic connections to regulate related gene expression, the release of trophic factors and changes in cerebral blood flow [11]. Neuroplasticity has been shown to underpin the recovery of cognitive function [12, 13]. As modern rehabilitation is moving towards community-based and home rehabilitation, the efficacy of non-invasive neuromodulation techniques in the later stages of VCI rehabilitation is expected.

At this stage, the most common non-invasive neuromodulation techniques include Transcranial Magnetic Stimulation (TMS), Transcranial Direct Current Stimulation (tDCS) and Transcutaneous Vagus Nerve Stimulation (TVNS), all of which can promote neuroplasticity. Repetitive Transcranial Magnetic Stimulation (rTMS), which involves repeated TMS stimulation at a target, can excite or inhibit cortical electrical activity through magnetic field stimulation depending on the different parameters [14], which has been widely used in cognitive-related rehabilitation [15, 16]. On the other hand, tDCS has also been shown to improve cognition. It is generally accepted that the tDCS anode increases the excitability of the subcortical layers [17] and can facilitate cognitive recovery by stimulating striatal dopamine release [18]. TVNS is a non-invasive vagal modulation technique that has been promoted in recent years,. It has been shown to produce vagally mediated pathways similar to those of invasive vagal electrical stimulation, including mainly transcutaneous auricular vagus nerve stimulation and transcutaneous cervical vagus nerve stimulation. Recent studies have shown that TVNS can improve cognitive function [19]. The mechanism of TVNS is unclear and may be related to the induction of NE and GABA. All three of these non-invasive neuromodulation techniques have been used in clinical VCI rehabilitation, and all have some theoretical basis and clinical efficacy. A network meta-analysis of non-invasive neuromodulation techniques for intervention in Alzheimer’s disease and mild cognitive impairment noted that HFrTMS was superior to atDCS in improving overall cognitive function [20]. In another systematic evaluation and meta-analysis of noninvasive neuromodulation techniques to intervene in behavioral and psychological symptoms of dementia, it was noted that pharmacological treatment had significant side effects, non-pharmacological treatment with rTMS was effective and safely tolerated, and the efficacy of tDCS was inconclusive [21]. In a network meta-analysis of similar Chinese and Western rehabilitation tools for intervention of post-stroke motor dysfunction, rTMS was found to be superior to tDCS and conventional therapy in improving Fugl Meyer Assessment (FMA) scale scores [22]. The network meta-analysis is expected to include all clinical studies on TMS, tDCS and TVNS interventions in VCI in the future to conduct a network meta-analysis of all relevant clinical data available. We hope to provide further scientific evidence and data which can support the application of non-invasive neuromodulation techniques in the rehabilitation of clinical VCI patients.

## Materials and methods

The network meta-analysis is conducted following the PRISMA Statement and Cochrane Reviews Handbook 5.1 for protocol development and study evaluation. In addition, the network meta-analysis has been registered on the international perspective systematic review register (PROSPERO): CRD42022308580.

### Inclusion criteria

#### Study type

The included studies are all randomised controlled trials with or without blinding and allocation concealment, and the language of the included studies are restricted to English or Chinese.

#### Inclusion

All patients are over 18 years of age and meet the diagnostic criteria for VCI with appropriate imaging bases such as CT or MRI, and their age, gender, race, disease duration, weight and education level will be not restricted.

#### Interventions

Major interventions are non-invasive neuromodulation techniques, including TMS, rTMS, tDCS, transcutaneous auricular vagus nerve stimulation (TaVNS) and transcutaneous cervical vagus nerve stimulation (TcVNS), without limiting their stimulation parameters and stimulation sites, may be combined with relevant drugs.

#### Control group

Either sham stimulation, placebo, medication, blank group, or interventions that include non-invasive neuromodulation techniques different from those used in the treatment group.

#### Outcome indicators

The primary outcome indicator is the total effective rate, and secondary outcome indicators include MoCa scale scores and MMSE scale scores. Studies containing any of these outcome indicators will be included in this systematic review.

### Exclusion indicators

Inclusion of patients with other malignant diseases.

Non-randomised controlled trials, animal studies, pathology reports, expert experience, conference papers.

Studies with incomplete data.

Repeated published studies.

### Data sources and search strategy

The network meta-analysis will search eight databases, including PubMed, Embase, Cochrane Library, Web of Science, China Knowledge Infrastructure Library (CNKI), China Biology Medicine disc (CBM)), Wanfang Data Knowledge Service Platform, and Vipshop Journal Service Platform (VIP). The search period is from the establishment of the database to January 30, 2022. The keywords include VCI, MCI, VaD, AD, TMS, rTMS, tDCS, TaVNS, TcVNS, vagus nerve and related terms. Table 1 summarized the search details for the relevant terms on PubMed. Table 2 is a summary of the characteristics of some of the published studies in relation to this research. For duplicate publications, we chose to include in this study the literature with the earliest publication date.

**Table 1:** Search strategy used in Pubmed database.

PubMed	
1	("Transcranial Magnetic Stimulation"[Mesh]) OR ("Transcranial Direct Current Stimulation"[Mesh]) OR ("Transcutaneous vagus nerve stimulation) "[Mesh])
2	(TMS) OR (rTMS) OR (Transcranial magnetic stimulation) OR (Magnetic Stimulation, Transcranial) OR (Magnetic Stimulations, Transcranial) OR (Stimulation, Transcranial Magnetic) OR (Stimulations, Transcranial Magnetic) OR (Transcranial Magnetic Stimulations) OR (Transcranial Magnetic Stimulation, Single Pulse) OR (Transcranial Magnetic Stimulation, Paired Pulse) OR (Transcranial Magnetic Stimulation, Repetitive) OR (Repetitive transcranial magnetic stimulation)
3	(tDCS) OR (Transcranial direct current stimulation) OR (Cathodal Stimulation Transcranial Direct Current Stimulation) OR (Cathodal Stimulation tDCS) OR (Cathodal Stimulation tDCSs) OR (Stimulation tDCS, Cathodal) OR (Stimulation tDCSs, Cathodal) OR (tDCS, Cathodal Stimulation) OR (tDCSs, Cathodal Stimulation) OR (Transcranial Random Noise Stimulation) OR (Transcranial Alternating Current Stimulation) OR (Transcranial Electrical Stimulation) OR (Electrical Stimulation, Transcranial) OR (Electrical Stimulations, Transcranial) OR (Stimulation, Transcranial Electrical) OR (Stimulations, Transcranial Electrical) OR (Transcranial Electrical Stimulations) OR (Anodal Stimulation Transcranial Direct Current Stimulation) OR (Anodal Stimulation tDCS) OR (Anodal Stimulation tDCSs) OR (Stimulation tDCS, Anodal) OR (Stimulation tDCSs, Anodal) OR (tDCS, Anodal Stimulation) OR (tDCSs, Anodal Stimulation) OR (Repetitive Transcranial Electrical Stimulation)
4	(Transcutaneous auricular vagus nerve stimulation) OR (Transcutaneous cervical vagus nerve stimulation) OR (Noninvasive vagus nerve stimulation) OR (tavns) OR (tcvns) OR (tvns) OR (nvns)
5	1 OR 2 OR 3 OR 4
6	(VCI) OR (Vascular cognitive impairment) OR (Mild Vascular cognitive impairment) OR ("Dementia, Vascular"[Mesh]) OR (VaD) OR (Vascular dementia) OR (Dementia, Vascular) OR (Dementias, Vascular) OR (Vascular Dementias) OR (Vascular Dementia, Acute Onset) OR (Acute Onset Vascular Dementia) OR (Subcortical Vascular Dementia) OR (Dementia, Subcortical Vascular) OR (Dementias, Subcortical Vascular) OR (Subcortical Vascular Dementias) OR (Vascular Dementia, Subcortical) OR (Vascular Dementias, Subcortical) OR (Arteriosclerotic Dementia) OR (Arteriosclerotic Dementias) OR (Dementia, Arteriosclerotic) OR (Dementias, Arteriosclerotic) OR (Binswanger Disease) OR (Disease, Binswanger) OR (Chronic Progressive Subcortical Encephalopathy) OR (Binswanger Encephalopathy) OR (Leukoencephalopathy, Subcortical) OR (Leukoencephalopathies, Subcortical) OR (Subcortical Leukoencephalopathies) OR (Encephalopathy, Subcortical Arteriosclerotic) OR (Encephalopathy, Subcortical, Chronic Progressive) OR (Subcortical Encephalopathy, Chronic Progressive) OR (Subcortical Leukoencephalopathy) OR (Subcortical Arteriosclerotic Encephalopathy) OR (Arteriosclerotic Encephalopathy, Subcortical) OR (Arteriosclerotic Encephalopathies, Subcortical) OR (Encephalopathies, Subcortical Arteriosclerotic)) OR (Subcortical Arteriosclerotic Encephalopathies) OR (Encephalopathy, Binswanger’s) OR (Binswanger’s Encephalopathy) OR (Encephalopathy, Binswangers) OR (Encephalopathy, Binswanger) OR (Encephalopathy, Chronic Progressive Subcortical)
7	(Randomized controlled trial)OR(randomized controlled trial as Topic)OR(controlled clinical trial)OR(controlled clinical trial)OR(randomized)
8	5 AND 6 AND 7

**Table 2:** Characteristics of the partially included studies.

Study	Design	Control group			Treatment group						Main findings	Follow-up
		N	Age	Intervention	N	Age	Intervention	N	Age	Intervention		
Wang 2016	RCT	30	69 ± 2.3	rTMS sham stimulation	30	71 ± 2.1	5Hz rTMS, 80% RMT, once a day for 4 weeks	30	70 ± 2.2	0.5Hz rTMS, 80% RMT, once a day for 4 weeks	The post-treatment MMSE scores for the control group and the two treatment groups were 14 ± 1.6, 27 ± 1.8, and 19 ± 1.6, and the MoCA scores were 16 ± 1.3, 28 ± 1.3, and 21 ± 1.3, respectively.	
Quan 2017	RCT	20	65.97 ± 5.71	rTMS sham stimulation	20	66.27± 5.35	10Hz rTMS, 80% RMT, 20min/time, 5 times/week	20	65.32 ± 5.41	1Hz rTMS, 80% RMT, 20min/time, 5 times/week	The post-treatment MMSE scores of the control group and the two treatment groups were 21.48±3.85, 25.89±3.96, and 25.97±3.82, respectively; the MoCA scores were 21.39±2.56, 26.72±3.81, and 26.67±3.79, respectively.	
Yu 2018	RCT	30	64.04 ± 1.83	No rTMS	30	63.77±2.05	15Hz rTMS, 5 times/week for 4 weeks				The mean change in the MoCA score in the treatment group was 19.97±3.87 vs 16.07±2.17 in the control group.	
Yu 2019	RCT	49	62.15 ± 13.49	No rTMS	51	65.13± 10.32	10 Hz rTMS, 80% RMT, 5x/week for 8 weeks				The post-treatment MMSE scores of the treatment group and the control group were 25.21±3.45 and 21.34±2.35, respectively; the MoCA scores were 24.52±4.32 and 20.24±2.35, respectively.	
Yin 2018	RCT	13	60.15 ± 10.29	rTMS sham stimulation	12	58.58± 11.98	10 Hz rTMS, 80% RMT, 5 times/week for 4 weeks				The mean change in the MoCA score in the treatment group was 20.92±5.05 vs 18±5.48 in the control group.	
Zhang 2019	RCT	30	55.11 ± 18.03	rTMS sham stimulation	30	58.44± 16.60	5 Hz rTMS, 80% RMT, 5 times/week for 4 weeks				The mean change in the MoCA score in the treatment group was 19.73±3.58 vs 17.5±4.57 in the control group.	
Li 2015	RCT	22	57.9 ± 4.3	No rTMS	23	58.6 ± 4.2	5 Hz rTMS, 80% RMT, 5 times/week for 4 weeks				The mean change in the MoCA score in the treatment group was 26.13±4.12 vs 23.36±4.71 in the control group.	
Wang 2016	RCT	20	58.3 ± 5.5	rTMS sham stimulation	20	57.9 ± 6.3	10 Hz rTMS, 80% RMT, 5x/week for 8 weeks				The mean change in the MoCA score in the treatment group was 25.25±3.98 vs 22.35±3.27 in the control group.	
Lu 2015	RCT	21	47.3 ± 11.8	rTMS sham stimulation	19	42.5 ± 12.3	1 Hz rTMS, 100% RMT, 5 times/week for 4 weeks				The mean change in the MoCA score in the treatment group was 22.16±3.948 vs 21.57±3.414 in the control group.	At 2 months after the end of stimulation, MoCA scores were still higher in the treatment group than in the sham-stimulation group, suggesting that the therapeutic effects of rTMS persisted until 2 months after the end of treatment.
Bao 2019	RCT	40	68.47 ± 6.87	No rTMS	40	68.74 ± 7.12	6 Hz rTMS, 120% RMT, 5 times/week for 8 weeks				The mean change in the MoCA score in the treatment group was 28.74±4.78 vs 23.74±4.27 in the control group.	
Zuo 2013	RCT	49	64.3 ± 7.8	No rTMS	53	62.6 ± 7.3	3 Hz rTMS, 80% RMT, 20 d of treatment total				The mean change in the MoCA score in the treatment group was 23.18± 3.47 vs 21.48 ± 3.56 in the control group.	
Wang 2014	RCT	56	55.4 ± 9.5	No rTMS	56	55.4 ± 9.5	0.5 Hz rTMS, 5 times/week for 4 weeks				The mean change in the MMSE score in the treatment group was 23±7 vs 17±7 in the control group.	
Tang 2015	RCT	30	60.9 ± 11.2	No rTMS	30	61.2 ± 10.8	5 Hz rTMS, 110% RMT, 6 times/week for 4 weeks				The mean change in the MoCA score in the treatment group was 24.47±1.41 vs 20.87±1.17 in the control group.	
Yuan 2018	RCT	22	68.68 ± 8.81	No rTMS	25	64.28 ± 6.59	10 Hz rTMS, 20 min/treatment for 20d				The mean change in the MMSE score in the treatment group was 21.27±3.06 vs 18.95±2.70 in the control group.	
Luo 2018	RCT	15	64.3 ± 5.1	No rTMS	15	65.8 ± 3.3	5 Hz rTMS, 5 times/week for 3 weeks				The post-treatment MMSE scores of the control group and the treatment group were 27.9±1.52 and 25.4±2.22, respectively; the MoCA scores were 26.3±2.79 and 22.8±2.52, respectively.	
Li 2019	RCT	25	49.63 ± 4.97	rTMS sham stimulation	25	49.79 ± 5.10	1 Hz rTMS, 80% RMT, 5 times/week				The post-treatment MMSE scores of the treatment group and the control group were 25.68±2.52 and 23.56±4.17, respectively; the MoCA scores were 26.98±3.47 and 21.87±3.29, respectively. In addition, the effectiveness rates of the observation and control groups were 92% and 68%, respectively.	
Ding 2019	RCT	15	53.53 ± 7.65	rTMS sham stimulation	15	54.33 ± 7.72	5 Hz rTMS10min followed by 1 Hz rTMS10min, 110% RMT, 6 times/week for 2 weeks				The post-treatment MMSE scores of the treatment group and the control group were 19.02±2.44 and 17.98±2.41, respectively; the MoCA scores were 17.74±3.16 and 16.8±2.87, respectively.	At 8 weeks after the end of stimulation, the MMSE scores and MoCA scores were still higher in the treatment group than in the sham-stimulation group, suggesting that the therapeutic effects of rTMS persisted until 2 months after the end of treatment.
Patikuli 2019	RCT	15	54.6 ± 9.90	No rTMS	15	65.07 ± 10.97	1 Hz rTMS10min followed by 10 Hz rTMS10min for 5d/week for 2 weeks				The post-treatment MMSE scores of the treatment group and the control group were 24.33±4.203 and 22.33±3.039, respectively; the MoCA scores were 16.50±4.171 and 17.00±3.817, respectively.	
Wu 2015	RCT	15	45–65	No rTMS	18	45–65	1 Hz rTMS for a total of 25 d of treatment				The post-treatment MMSE scores of the treatment group and the control group were 29.06±0.8 and 28.2±1.15, respectively; the MoCA scores were 24.67±2.85 and 23.2±2.21, respectively.	
Zheng 2017	RCT	30	61.97 ± 11.39	No rTMS	30	58.8 ± 13.54	20 Hz rTMS, 80% RMT, 5 times/week for 6 weeks				The post-treatment MMSE scores of the treatment group and the control group were 24.27±2.2 and 16.4±1.19, respectively; the MoCA scores were 25.7±1.6 and 22.93±2.05, respectively.	
Zhang 2015	RCT	10	57.9 ± 9.2	No rTMS	10	50.4 ± 8.8	rTMS, 5 times/week for 4 weeks				The mean change in the MMSE score in the treatment group was 20.1±2.17 vs 19.36±2.63 in the control group.	
Hu 2016	RCT	30	56.2 ± 10.9	No rTMS	30	57.5 ± 13.3	10 Hz rTMS, 5 times/week for 4 weeks				The post-treatment MMSE scores of the treatment group and the control group were 22.5±1.53 and 21.47±1.25, respectively; the MoCA scores were 23.47±1.53 and 20.73±1.55, respectively.	

### Study data extraction and quality evaluation

All study data extraction and quality analysis will be done by the two independent reviewers. Discrepancies between two reviewers will resolved through discussions with a third reviewer.

### Studies inclusion

**Fig 1:**
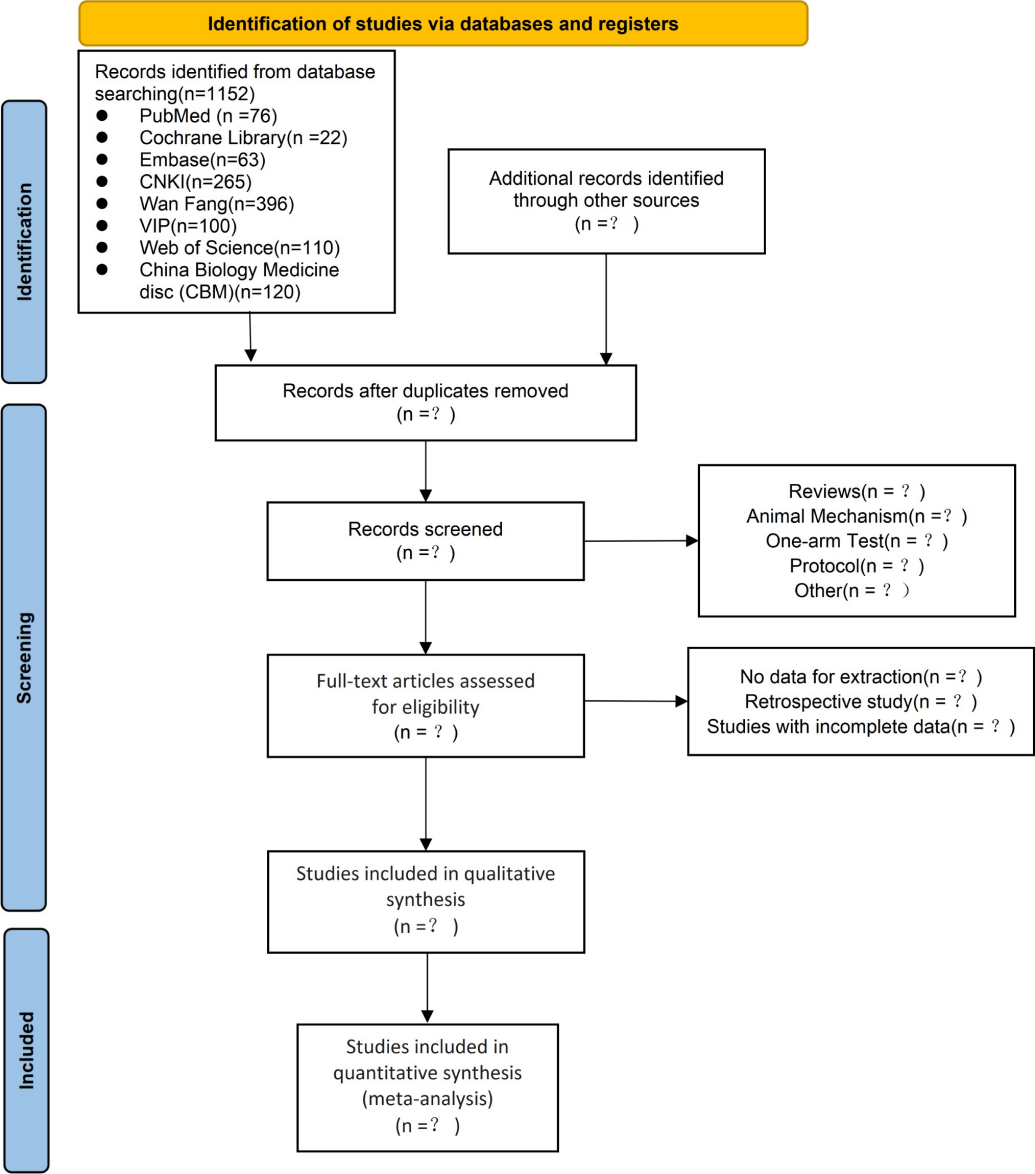
PRISMA flow chart of study and exclusion.

Two reviewers will search relevant studies individually based on the studies search strategy, and the results will be compared and supplemented after the search. The two reviewers will eliminate duplicate studies using the NoteExpress check function and read the title, abstract and keywords to eliminate studies which are not relevant to the network meta-analysis, read the full text according to the nadir criteria, and finally include the studies that meet the criteria in the study. We created a PRISMA flow chart to show the whole process Fig 1.

### Data extraction

Two reviewers independently should design a standardised data extraction form which contains basic information about the study (including first author, year of publication, nationality), essential demographic characteristics of the intervention population (including sample size, age, gender, diagnosis, duration of disease), study characteristics (including study type, grouping method, blinding, allocation concealment), intervention protocol (including intervention type, intervention parameters, intervention period), outcome indicators (including primary outcome indicators, secondary outcome indicators, follow-up), adverse effects. All outcome indicators are based on data at the end of the intervention and do not include follow-up data.

### Quality assessment

Two independent reviewers will evaluate the quality of the included studies using the Risk of bias tool(ROB2), containing the following entries:① Randomization process;② Deviations from intended interventions; ③ Missing outcome data;④ Measurement of the outcome; ⑤ Selection of the reported result); ⑥ Overall Bias. The above six items will be evaluated on a scale of "Low Risk", "Some concerns", and "High Risk". On the other hand, the PEDro scale will be used to evaluate the quality of the study, consisting of 11 items with a total score of 11. A score of 9–11 will be regarded as ’excellent’, 6–8 as ’good’, 3–5 as ’general’ and below 3 as ’bad’.

### Data analysis

#### Handling of missing data

For studies where data are not fully reported, the authors will be contacted by email in an attempt to obtain the complete raw data. If the amount of changes before and after the intervention are not fully reported, the mean and SD will be calculated manually using the Cochrane Handbook formulae based on the reported baseline and outcome data.

#### Classical meta-analysis

All included studies will be first subjected to classical meta-analysis using Review Manager program. The total effective rate is a dichotomous variable and its OR with 95% CI will be calculated; the remaining scale scores are continuous variables, so the standardised mean differences (SMDs) and corresponding 95% CI will be assessed. Heterogeneity will be assessed using the I^2^ test [23]. P <0.05 or I^2^ >50% will be considered high heterogeneity, and a random-effects model will be used; otherwise, we will use a fixed-effects model. Heterogeneity will be identified using sensitivity analysis or a subgroup analysis based on age, duration and aetiology of disease included in the study to determine the source of heterogeneity. If the source of heterogeneity cannot be determined, a descriptive analysis of the corresponding study will be performed. We will assess publication bias using funnel plots if the number of included studies exceeds 10 cases. The Egger regression test will assess the asymmetry generated by the funnel plot [24].

#### Reticulated meta-analysis

Network relationships will be plotted using Stata program. If the included studies are more than two-armed trials which will be split and reorganised into the corresponding two-armed trials [25]. The network meta-analysis will be undertaken in WinBUGS1.4.3 software, using the Bayesian Markov Chain Monte Carlo random effect mode [26]. The convergence of MCMC is expressed in terms of the scaled reduction parameter, with PSRF close to 1 being considered good model convergence and higher reliability [27]. Nodal splitting will be used to assess the consistency of direct and indirect evidence in the closed loop [28]. Finally, each outcome indicator included in the studies will be used to generate cumulative ranking curves using the Stata program to predict and rank the efficacy of the various interventions [29].

#### Grading the quality of evidence

Two reviewers will assess the quality of evidence for the study using the recommended assessment tool and categorise the evidence to four levels as ’high’, ’moderate’, ’low’ and ’very low’. Disagreement will be solved by a third reviewer.

#### Patient and public involvement

No patients or public will be involved in this study.

## Discussion

The trend of modern rehabilitation is not only towards better rehabilitation outcomes, but also the need for non-invasive, simple, inexpensive, home or community-based rehabilitation. Non-invasive neuromodulation technology, as a cutting-edge medical technology whose mechanism of action to promote neuroplasticity, should not be overlooked in the rehabilitation of VCI. Although some of the access mechanisms are not yet clear, they have shown some efficacy in the clinical rehabilitation of VCI. In the network meta-analysis, we will systematically compare the efficacy and safety of various non-invasive neuromodulation techniques and corresponding drug combinations in the rehabilitation of VCI. The results of this network meta-analysis will synthesise direct and indirect evidence [30] to provide a preferred protocol for the intervention of non-invasive neuromodulation techniques in the rehabilitation of VCI [31]. Therefore, we hope that a rigorous network meta-analysis will provide more evidences to support the clinical application of non-invasive neuromodulation interventions in VCI. However, there are some potential weaknesses in the network meta-analysis: on the one hand, the lack of quality of the original studies directly affects the final effect of the network meta-analysis; on the other hand, the difference in the dose of medication administered to the patients and the corresponding parameters in the non-invasive neuromodulation technique may also lead to differences in the outcome. Therefore, we will strictly control the quality of the included studies to provide reliable clinical evidence to support the development of non-invasive neuromodulation techniques in the rehabilitation of VCI.

## Supporting information

S1 File PRISMA-P (preferred reporting items for systematic review and meta-analysis protocols) 2015 checklist. (PDF)
