# Supplementary material for: Effect of non-invasive neuromodulation techniques on vascular cognitive impairment: A Bayesian network meta-analysis protocol
Source: PLoS One. 2024 Jan 4;19(1):e0284447. doi: 10.1371/journal.pone.0284447 (PMC10766188; doi:10.1371/journal.pone.0284447)
Supplement: Attachment — Submitted filename: Response to Reviewers.docx [file pone.0284447.s002.docx]

March 23, 2023

Dear reviewers and editors,

Allow me to begin by sending you my most sincere greetings. I am honoured to receive suggestions from you, which have been most helpful to my manuscript. Based on the comments we received, we have further sorted out and revised my manuscript and am submitting the revised manuscript and the revision process to you.

Response to Reviewers:

**Author Response:**Thank you for your suggestion. We have renamed and uploaded the revised files as you suggested to make them compliant with the journal requirements.

Response to Journal:

1 Please ensure that your manuscript meets PLOS ONE's style requirements, including those for file naming. The PLOS ONE style templates can be found at

https://journals.plos.org/plosone/s/file?id=wjVg/PLOSOne_formatting_sample_main_body.pdf and

<https://journals.plos.org/plosone/s/file?id=ba62/PLOSOne_formatting_sample_title_authors_affiliations.pdf>

**Author Response:**We have ensured that our files comply with PLOS ONE requirements including the naming of the files.

2. Our internal editors have looked over your manuscript and determined that it is within the scope of our Reproducibility and Replicability in Neuroscience and Mental Health Research Call for Papers. The Collection will encompass a diverse and interdisciplinary set of protocols and research articles adhering to transparent and reproducible reporting practices in the areas of clinical psychology, psychiatry, mental health, and neuroscience. Additional information can be found on our announcement page: https://collections.plos.org/call-for-papers/reproducibility-and-replicability-in-neuroscience-and-mental-health-research/. If you would like your manuscript to be considered for this collection, please let us know in your cover letter and we will ensure that your paper is treated as if you were responding to this call. If you would prefer to remove your manuscript from collection consideration, please specify this in the cover letter.

**Author Response:**Thank you for your suggestions.

3. Thank you for stating the following financial disclosure:

“No”

At this time, please address the following queries:

a)        Please clarify the sources of funding (financial or material support) for your study. List the grants or organizations that supported your study, including funding received from your institution.

b)        State what role the funders took in the study. If the funders had no role in your study, please state: “The funders had no role in study design, data collection and analysis, decision to publish, or preparation of the manuscript.”

c)        If any authors received a salary from any of your funders, please state which authors and which funders.

d)        If you did not receive any funding for this study, please state: “The authors received no specific funding for this work.”

Please include your amended statements within your cover letter; we will change the online submission form on your behalf.

**Author Response:**This study was supported by the National Natural Science Foundation of China, Youth Science Foundation Project (No.81704148), the Tianjin Postgraduate Research Innovation Project (No.2020YJSB197) and the Tianjin Appropriate Technology Promotion Project for Chinese Medicine Rehabilitation Services. This study involved two funders,Yu Wang and Baomin Dou, who provided the main financial support in the study. The funding received for this study is mainly for the preliminary paper search, which costs about $80, and the post-paper publication and other expenses are funded according to the actual situation.

4. Please include captions for your Supporting Information files at the end of your manuscript, and update any in-text citations to match accordingly. Please see our Supporting Information guidelines for more information: <http://journals.plos.org/plosone/s/supporting-information>.

**Author Response:** We have added titles to the manuscript's supplementary information file as you suggested.

5. We note that this manuscript is a systematic review or meta-analysis; our author guidelines therefore require that you use PRISMA guidance to help improve reporting quality of this type of study. Please upload copies of the completed PRISMA checklist as Supporting Information with a file name “PRISMA checklist”.

**Author Response:** We have followed your suggestion to upload the complete PRISMA checklist.

6. Please review your reference list to ensure that it is complete and correct. If you have cited papers that have been retracted, please include the rationale for doing so in the manuscript text, or remove these references and replace them with relevant current references. Any changes to the reference list should be mentioned in the rebuttal letter that accompanies your revised manuscript. If you need to cite a retracted article, indicate the article’s retracted status in the References list and also include a citation and full reference for the retraction notice.

**Author Response:** We made overall changes to the reference format and added three new references, 20, 21, and 22, details can be found on pages 15-19.

Response to Editor

1 Background: As the authors point out several different types of non-invasive nerve stimulation technology has been employed over the last 2 decades for treatment of vascular cognitive impairment. As such, a network meta-analysis framework appears warranted. However, the introduction sections of the manuscript may be improved by adding additional background on the different approaches, including references of any previous reviews on the subject, citation of any previous head-to-head comparisons between nerve stimulation technologies (and any other pharmacological treatments), and example use of network meta-analysis in other closely related interventional examples (that may shed further light on the authors hypothesis that a network meta-analysis will reveal additional support (that is not now currently apparent without it) for non-invasive neuromodulation in VCI).

**Author Response:** We have added to the introduction section as you suggested, which can be found on pages 5 and 6.

2 Title: The authors use non-invasive neuromodulation in much of the text but chose “nerve regulation technology” in the title. “Neuromodulation” appears to be the preferred term in the field; should the authors change the title and/or have a few sentences in the background defining “neuromodulation” vs. “nerve regulation”.

**Author Response:** We have changed the title from "noninvasive nerve regulation technology" to "non-invasive neuromodulation techniques" as you suggested, see page 1.

3 Outcome indicators in Inclusion Criteria: It may be of interest here to also include other outcome indicators used in the field that were excluded; perhaps this could be a section of “exclusion criteria” (as it appears that outcomes other than MoCa scale and MMSE scale are being excluded?)

**Author Response:** Thank you for your suggestion. Regarding the issue of outcome indicators, the outcome indicators of the inclusion criteria in our manuscript were divided into primary outcome indicators and secondary outcome indicators, with the primary outcome indicator being the efficiency rate and the secondary outcome indicators being the MMSE and MoCa scales, that is, the outcome indicators in the final included literature must contain the efficiency rate, but not all included literature contain the MMSE or MoCa scales, so the secondary outcome indicators are not necessary for inclusion, and the exclusion criteria are not considered as not containing the MMSE and MoCa scales.

4 Data Extraction: Would it be possible for this manuscript to start with a baseline data extraction form? While it will/must be refined during the project work; enough is known about the studies now to show a starting point?

**Author Response:** We have designed a baseline data extraction form based on your suggestion, but since we are currently screening eligible literature based on inclusion and exclusion criteria, we have not yet fully extracted the data. Details can be found in Table 3.

5 Meta Analysis Methods: Are there any direct comparisons between these approaches/interventions currently in literature? Have any of the neuro-modulation technologies been compared to other pharmaceutical approaches? If either of these are “Yes”, then the current inclusion/exclusion criteria may be eliminating potentially valuable direct and/or indirect comparators between the approaches? Some sort of explanation of the expected treatment geometry should be discussed, in this context (i.e., some incoming understanding of the # of studies expected and/or types of comparison studies available)?

**Author Response:** Thank you for your suggestion. There is no direct comparison of TMS, tDCS, and TVNS in the literature on non-invasive neuromodulation techniques for the treatment of vascular cognitive disorders, but there is a direct comparison of TMS and tDCS for the treatment of Alzheimer's disease and other dementias, and a meta-analysis has also mentioned the advantages and disadvantages of pharmacological and non-pharmacological therapies and the efficacy of non-pharmacological therapies (TMS and tDCS) comparison. As to whether the current inclusion and exclusion criteria may preclude valuable direct or indirect comparisons between these approaches, we will detail the course of treatment and outcomes for each intervention in the baseline data extraction table, as well as comparisons between the two interventions, if any, and each outcome indicator included in the study will be used to generate cumulative ranking curves in the final network meta-analysis. The Stata program was used to predict and rank the effects of the various interventions, as described on page 12.

6 Meta Analysis Methods: For these new technologies, there is the potential for a large number of “small n studies”, and insufficiently powered network analysis. What type of sample size and power considerations will be employed in this study?

**Author Response:** Inadequate sample size in meta-analysis can lead to low statistical power, which can lead to possible false negatives or false positives in the estimated effect values, for which we will provide the following explanation.

Regarding the sample size, the number of studies and the sample size of each study determine whether we want to include them or not. In layman's terms, if the number of studies is relatively large, studies with small samples are not included, and if the number of studies is small, studies with small samples should still be considered. Also the effect size should be considered in the analysis, and if the effect size of the small sample studies is the same as the effect size of the large sample studies, they can be combined in the analysis. If the effect size of the small sample differs significantly from that of the large sample, the combined effect size may not explain the true clinical effect. Therefore, in such cases, a detailed description of the large sample study alone would be more clinically valuable.

In recent years, with the development of the GRADE evidence rating system, the GRADE working group has made corresponding provisions for the evaluation of the precision of the results of network meta-analysis, proposing the calculation of equivalent sample size. Therefore, the sample size and statistical power can be calculated according to the corresponding formula.

**7** Meta Analysis Methods: Again, are there direct comparison studies and/or indirect comparators (such as comparisons of multiple neuromod techniques to standard of care or drug) available? If so, what statistical methods will be utilized to detection heterogeneity and/or incoherence in the data sets?

**Author Response:** In the literature on non-invasive neuromodulation techniques for vascular cognitive impairment, there is no direct comparison of TMS, tDCS and TVNS, but there is a direct comparison of TMS and tDCS for Alzheimer's disease and other dementias, as well as a meta-analysis mentioning the advantages and disadvantages of pharmacological and non-pharmacological therapies and a comparison of the efficacy of non-pharmacological therapies (TMS and tDCS).

All included studies will be first subjected to classical meta-analysis using Review Manager program. The total effective rate is a dichotomous variable and its OR with 95% CI will be calculated; the remaining scale scores are continuous variables, so the standardised mean differences (SMDs) and corresponding 95% CI will be assessed. Heterogeneity will be assessed using the I^2^ test. P <0.05 or I^2^ >50% will be considered high heterogeneity, and a random-effects model will be used; otherwise, we will use a fixed-effects model. Heterogeneity will be identified using sensitivity analysis or a subgroup analysis based on age, duration and aetiology of disease included in the study to determine the source of heterogeneity. If the source of heterogeneity cannot be determined, a descriptive analysis of the corresponding study will be performed，as described on page 12.

**8** Meta Analysis Methods: Is there sufficient comparisons to placebo across these non-invasive neuromod techniques yet to enable a meta analysis to be effective? Can lack of this information lead to eventual misinterpretations? Will there be plain treatment rankings and probabilities in the outcome tables; how will the author ensure that there aren’t potentially miss-leading?

**Author Response:** Among these non-invasive neuromodulation techniques, monotherapy is often compared with sham stimulation or placebo, and direct comparisons between two neuromodulation techniques are also available, thus allowing network meta-analysis to be performed effectively. The lack of such information may increase the risk of error in the results.

Information on the treatment ranking and efficiency of each neuromodulation technique will be presented in the table.The manuscript mentions that the outcome metrics will generate cumulative ranking curves using the Stata program, details of which can be found on page 12.

Regarding how to ensure that there is no potential for misinformation, this relates to how several interventions are compared with each other, whether directly or indirectly, and in more detail, is closely related to sample size, effect size and statistical efficacy. That is, the sample size is calculated, including the required sample size and the valid or equivalent sample size for the network meta-analysis, and the precision of the effect estimates is evaluated. The details of the network meta analysis can be found on pages 11 and 12.

**9** What if the availability of current data does not currently warrant a network meta-analysis? How will the authors conclude this, and how will it be reported? Or, should the authors be expected to perform some initial analysis that concludes one is warranted prior to publication of the manuscript? This is the key question that must be resolved with some significant revisions prior to publication.

**Author Response:** Thank you for your suggestion. If the evidence network for an outcome is not well linked (e.g., no closed loop between interventions), we will report the results of the paired meta-analysis. If the study team judges a study to be too different from the remaining studies reporting a common intervention based on clinical characteristics, we will perform an NMA without that study and explore possible options for sensitivity analyses. We will also have conducted sensitivity analyses using alternative associations between baseline and mean values at follow-up for each outcome. Whenever feasible, we also plan to explore the impact of subgroup analysis of each factor on the results.

Given that the study is still ongoing, we have appropriate responses for all possible outcomes in the study and will also further analyze and discuss the results at a later stage.

Finally, I would like to thank the reviewers and the editor for your suggestions on my manuscript again. If there are any further problems with the manuscript, we look forward to hearing from you.

Best Regards.

[Long Yan, Linna Wu, Hong Li, Yulin Qian, Meng Wang, Yu Wang, Baomin Dou, Tao Yu]

[1 Tianjin University of traditional Chinese Medicine;2 First Teaching Hospital of Tianjin University of Traditional Chinese Medicine;3 Tianjin University of Traditional Chinese Medicine]

[[505781130@qq.com]](mailto:[15222817069@163.com])
